# Metabolic Mechanisms of Neutrophil Phagocytic Activity in Patients with Widespread Purulent Peritonitis Bacterial Peritonitis Before and After Surgery

**DOI:** 10.3390/ijms27115128

**Published:** 2026-06-05

**Authors:** Andrey A. Savchenko, Dmitry Kudlay, Ivan I. Gvozdev, Elena N. Anisimova, Dmitry V. Cherdantsev, Igor Kudryavtsev, Artem Rubinstein, Anna An. Starshinova, Alexandr Borisov

**Affiliations:** 1 Federal Research Center «Krasnoyarsk Science Center», Siberian Branch, Russian Academy of Sciences, Scientific Research Institute of Medical Problems of the North, 660036 Krasnoyarsk, Russia; 2 NRC Institute of Immunology FMBA of Russia, 115478 Moscow, Russia; 3 Department of Pharmacology, Institute of Pharmacy, I.M. Sechenov First Moscow State Medical University, 119435 Moscow, Russia; 4 Faculty of Bioengineering and Bioinformatics, Lomonosov Moscow State University, 119991 Moscow, Russia; 5 Department of Hospital Surgery Named After Professor A.M. Dykhno, Krasnoyarsk State Medical University, 660022 Krasnoyarsk, Russia; 6 Institute of Experimental Medicine, 197376 Saint Petersburg, Russia; 7 Almazov National Medical Research Center, 197341 Saint Petersburg, Russia; 8 Faculty of Mathematics and Computer Science, Saint Petersburg State University, 199034 Saint Petersburg, Russia

**Keywords:** anaerobic and aerobic metabolism, synthetic processes, bacterial peritonitis, glucose-6-phosphate dehydrogenases, immunometabolism, neutrophils, oxidative stress, phagocytic activity, postoperative outcome, prognostic biomarkers, reverse neutrophil migration

## Abstract

Bacterial peritonitis (BP) remains a significant clinical challenge due to its high risk of multiple organ failure and associated mortality. Neutrophils are central effectors of innate immunity, and their functional activity and metabolism may influence the progression and outcomes of immunoinflammatory diseases. To investigate the phagocytic activity and intracellular metabolic profiles of neutrophils in patients with BP and to evaluate their relationship with postoperative clinical outcomes, 51 patients with BP (23 men, 28 women; mean age 49.6 ± 9.4 years) were examined. Blood samples were collected preoperatively and on postoperative day 7. Phagocytic activity of total, actively, and weakly phagocytic neutrophils was assessed using flow cytometry. Intracellular activity of NAD(P)- and NAD(P)H-dependent oxidoreductases and dehydrogenases was measured by bioluminescence. Patients were stratified according to postoperative outcome: favorable (*n* = 32) or unfavorable (*n* = 19). Seventy healthy individuals served as controls. Preoperatively, the proportion and phagocytic activity of neutrophils were markedly elevated in all patients. Postoperatively, the proportion of phagocytosing neutrophils remained high; however, phagocytic activity increased in patients with favorable outcomes but decreased to control levels in those with unfavorable outcomes. Neutrophil metabolism before surgery exhibited activation of both anaerobic and aerobic pathways, accompanied by reduced glucose-6-phosphate dehydrogenase activity. Postoperative metabolic adaptations differed according to outcome: patients with favorable outcomes demonstrated normalization of energy metabolism, whereas patients with unfavorable outcomes exhibited enhanced anaerobic metabolism, persistent aerobic activity, increased substrate flux towards glutamate/glutamine synthesis, and intensified lipid peroxidation. Phagocytic activity and metabolic profiles of neutrophils in BP are outcome-dependent. Effective postoperative anti-inflammatory responses, including reverse migration of activated neutrophils, are associated with favorable outcomes, whereas persistent metabolic activation and oxidative stress correlate with unfavorable prognosis. Neutrophil functional and metabolic parameters may serve as prognostic biomarkers and potential targets for therapeutic modulation in BP.

## 1. Introduction

Despite the wide range of intensive care modalities available, bacterial peritonitis (BP) remains a major challenge in modern healthcare owing to the persistently high risk of multiple organ dysfunction syndrome (MODS) and, consequently, high mortality rates [1,2].

An essential component of postoperative management in patients with BP is the objective assessment of the patient’s condition, which enables prediction of the disease course, the likelihood of complications, and the risk of an unfavorable outcome. To address this task, a number of scoring systems and prognostic indices are widely used, including APACHE, SAPS, SOFA, and others [3].

However, the clinical course and outcome of BP are determined not only by the severity of the underlying pathological process, the adequacy of surgical intervention, and pharmacological therapy, but also by alterations occurring within the immune system [4,5]. In particular, a study by Menz et al. (2021) demonstrated that surgically induced immune dysfunction in an experimental model led to increased severity of peritonitis and higher mortality rates [6].

It should be noted that the main therapeutic strategy in the postoperative management of patients with BP is based on the use of antibacterial agents, which exert not only direct antimicrobial effects but may additionally modulate the functional activity of immune cells [7,8].

At the early stages of BP immunopathogenesis, neutrophil granulocytes are actively involved in the development of both local and systemic inflammatory responses [5,9,10]. Neutrophils are not only effector cells responsible for phagocytosis, the synthesis of cytotoxic molecules, and the formation of neutrophil extracellular traps (NETs), but are also capable of synthesizing a wide range of receptors and cytokines, thereby participating in the regulation of adaptive immune responses [11,12,13].

In particular, a study by Fukui et al. demonstrated that during the development of peritonitis, neutrophil granulocytes actively synthesize and secrete interleukin-1β (IL-1β) [9]. Moreover, under various immunoinflammatory conditions, neutrophils are capable of producing IL-6, IL-16, and IL-18, making a substantial contribution to the development of cytokine storm [14].

The functional activity of neutrophils cannot be realized without profound reprogramming of intracellular metabolic processes. It has been shown that various immunopathological conditions are associated with marked dysregulation of intracellular metabolism in neutrophil granulocytes [15,16,17]. In a review by Curi et al. (2020), the pivotal role of energy-producing and biosynthetic pathways in supporting neutrophil migration, phagocytosis, and reactive oxygen species generation was emphasized [18].

Phelan and Sheedy (2021) demonstrated that neutrophil phagocytic activity is dependent on the activity of the key glycolytic enzyme phosphofructokinase [19]. In our previous work, we established the prognostic significance of NAD-dependent substrate efflux from tricarboxylic acid cycle reactions in neutrophils of patients with BP prior to surgery with respect to the risk of postoperative sepsis development [20].

Given the highly informative value of metabolic parameters in characterizing the functional state of neutrophils, investigation of their metabolic profiles allows for a deeper understanding of the mechanisms underlying immune dysfunction, facilitates assessment of disease prognosis, and provides a rationale for optimizing postoperative therapeutic strategies in patients with BP. However, enzyme activity measurements do not directly reflect metabolic fluxes and should therefore be interpreted cautiously. Accordingly, the present study aimed to investigate neutrophil phagocytic activity and metabolic characteristics in relation to the clinical outcome of diffuse purulent peritonitis.

## 2. Results

Analysis of the phagocytic activity of the total circulating neutrophil fraction demonstrated that, already in the preoperative period, patients with bacterial peritonitis (BP) exhibited increased phagocytic index (PI) and phagocytic number (PN) compared with control values, irrespective of disease outcome (Table 1). On postoperative day 7, PI remained markedly elevated in all examined patients, regardless of outcome. However, PN showed opposite trends depending on the clinical course. In patients with a favorable outcome, postoperative phagocytic activity increased further, whereas in patients with an unfavorable outcome, PN decreased compared with preoperative values and approached the control range. The control group parameters were determined once.

In patients with a favorable outcome, PN increased more than two-fold compared with baseline values and exceeded control levels by more than five-fold. In contrast, in patients with an unfavorable outcome, PN decreased almost two-fold relative to baseline and fell within the control range.

Based on PN values, the total fraction of phagocytosing neutrophils (100%) was subdivided into actively and weakly phagocytic cells [20,21,22]. The proportion of actively phagocytic neutrophils (PI) in patients with DPP during the preoperative period significantly exceeded control values and did not differ according to disease outcome (Table 2). At the same time, PN was more than two-fold higher than control values in patients with a favorable outcome and 2.7-fold higher in those with an unfavorable outcome.

Following surgery, both PI and PN of actively phagocytic neutrophils increased further in patients with a favorable outcome compared with baseline values [23,24]. Conversely, patients with an unfavorable outcome exhibited a pronounced decline in both parameters.

Accordingly, the proportion of weakly phagocytic neutrophils in patients with BP during the preoperative period was reduced compared with controls and did not differ depending on disease outcome (Table 3). Despite this, this neutrophil subpopulation demonstrated significantly higher PN values than those observed in the control group, with no differences between outcome groups.

In the postoperative period, patients with a favorable outcome showed a further reduction in PI of weakly phagocytic neutrophils, both relative to baseline values and the control range. In contrast, patients with an unfavorable outcome demonstrated an increase in PI compared with baseline, reaching values within the control range. PN values in both outcome groups did not change significantly after surgery compared with preoperative levels.

**Figure 1:**
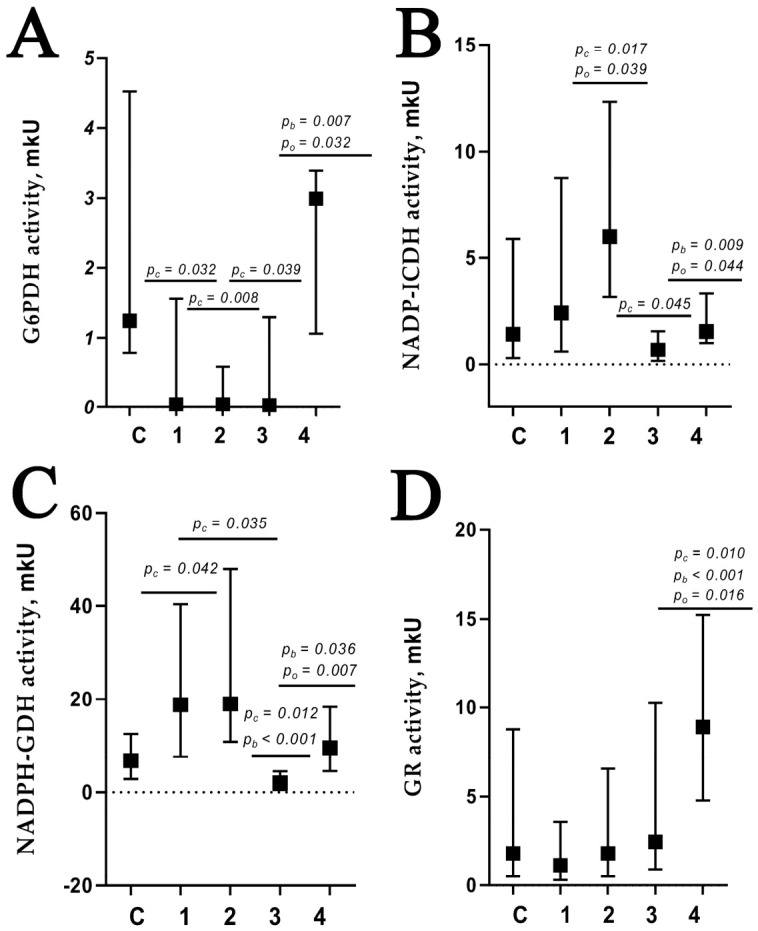
Activity of NADP- and NADPH-dependent oxidoreductases in patients with bacterial peritonitis (BP) before and after surgery with favorable and unfavorable outcomes. Along the abscissa axis: C—control; 1—patients with BP and a favorable outcome before surgery; 2—patients with BP and an unfavorable outcome before surgery; 3—patients with BP and a favorable outcome after surgery; 4—patients with BP and an unfavorable outcome after surgery. *p*_c_—statistically significant differences compared with control values; *p*_b_—differences compared with baseline (preoperative) values; *p*_o_—differences between patients with favorable and unfavorable outcomes. (**A**) Glucose-6-phosphate dehydrogenase (G6PDH) activity, mkU (**B**) Activity of NADP-dependent isocitrate dehydrogenase (NADP-ICDH) dehydrogenase, mkU (**C**) Activity of NADPH-dependent glutamate dehydrogenase (NADPH-GDH), mkU (**D**) Glutathione reductase (GR) activity, mkU.

Assessment of NADP- and NADPH-dependent oxidoreductase activity in neutrophils revealed that glucose-6-phosphate dehydrogenase (G6PDH) activity in patients with BP was significantly reduced in the preoperative period compared with control values and did not differ according to disease outcome (Figure 1A). In the postoperative period, G6PDH activity in neutrophils of patients with a favorable outcome remained largely unchanged and thus persisted at a reduced level relative to controls. In contrast, patients with an unfavorable outcome exhibited a marked postoperative increase in G6PDH activity, reaching values within the control range.

Preoperative NADP-dependent isocitrate dehydrogenase (NADP-ICDH) activity in neutrophils of patients with a favorable outcome corresponded to the control range (Figure 1B). Conversely, patients with an unfavorable outcome demonstrated significantly elevated activity of this enzyme compared with both control values and levels observed in the favorable outcome group. After surgery, NADP-ICDH activity decreased in both groups: in patients with a favorable outcome, enzyme activity fell below the control range, whereas in those with an unfavorable outcome, it declined to control levels but remained significantly higher than in patients with a favorable outcome.

In the preoperative period, NADPH-dependent glutamate dehydrogenase (NADPH-GDH) activity in neutrophils of patients with BP significantly exceeded control values, irrespective of disease outcome (Figure 1C). Postoperatively, intracellular enzyme activity declined markedly in both patient groups compared with baseline. However, whereas NADPH-GDH activity in patients with a favorable outcome fell below the control range, in patients with an unfavorable outcome, it remained within the control range and exceeded the levels observed in the favorable outcome group. Glutathione reductase (GR) activity in neutrophils of patients with a favorable outcome of BP both before and after surgery corresponded to control values (Figure 1D). In patients with an unfavorable outcome, GR activity in the preoperative period was also comparable to the control level; however, after surgery, it increased significantly relative to baseline values and exceeded the activity observed in both the control group and patients with a favorable outcome.

**Figure 2:**
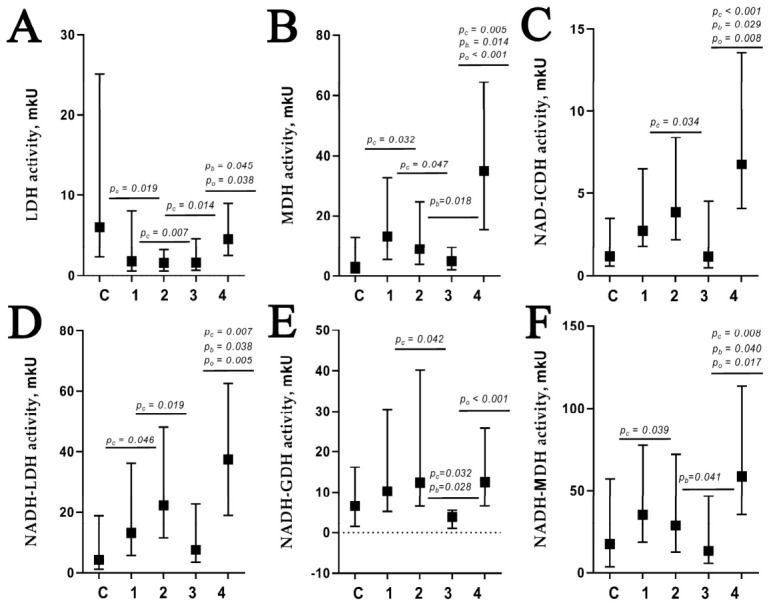
Activity of NAD- and NADH-dependent dehydrogenases in patients with bacterial peritonitis (BP) before and after surgery with favorable and unfavorable outcomes. Along the abscissa axis: C—control; 1—patients with BP and a favorable outcome before surgery; 2—patients with BP and an unfavorable outcome before surgery; 3—patients with BP and a favorable outcome after surgery; 4—patients with BP and an unfavorable outcome after surgery. *p*_c_—statistically significant differences compared with control values; *p*_b_—differences compared with baseline (preoperative) values; *p*_o_—differences between patients with favorable and unfavorable outcomes. (**A**) Activity of NAD-dependent reaction of lactate dehydrogenase (LDH), mkU (**B**) Activity of NAD-dependent reaction of malate dehydrogenase (MDH), mkU (**C**) Activity of NAD-dependent isocitrate dehydrogenase (NAD-ICDH), mkU (**D**) Activity of NADH-dependent reaction of lactate dehydrogenase (NADH-LDH), mkU (**E**) Activity of NADH-dependent reaction of malate dehydrogenase (NADH-MDH), mkU (**F**) Activity of NADH-dependent glutamate dehydrogenase (NADH-GDG), mkU.

Assessment of NAD- and NADH-dependent dehydrogenase activity in circulating neutrophils revealed that lactate dehydrogenase (LDH) activity in patients with BP was significantly reduced in the preoperative period compared with control values and was independent of disease outcome (Figure 2A). After surgery, LDH activity in patients with a favorable outcome remained unchanged, whereas in those with an unfavorable outcome it increased relative to baseline, reached the control range, and exceeded the levels observed in patients with a favorable outcome at this stage of observation.

Malate dehydrogenase (MDH) activity in neutrophils of both patient groups exceeded control values prior to surgery, while postoperative changes differed depending on disease outcome (Figure 2B). In patients with a favorable outcome, MDH activity decreased relative to baseline to values within the control range. In contrast, patients with an unfavorable outcome demonstrated a significant postoperative increase in MDH activity, exceeding both control values and levels observed in patients with a favorable outcome at the final stage of the study.

Elevated NAD-dependent isocitrate dehydrogenase (NAD-ICDH) activity in circulating neutrophils during the preoperative period was observed exclusively in patients with an unfavorable outcome of BP (Figure 2C).

Based on the data obtained, presented in Figure 2, the preoperative activity of this enzyme was elevated relative to control values only in patients with an unfavorable outcome. Postoperative activity of this enzyme was elevated only in patients with an unfavorable outcome relative to control and baseline values, as well as in patients with a favorable outcome.

In patients with a favorable outcome, postoperative NAD-ICDH activity remained within the control range. Conversely, in patients with an unfavorable outcome, enzyme activity increased markedly after surgery compared with baseline values and exceeded the activity levels observed in both the control group and patients with a favorable outcome at the corresponding time point.

NADH-dependent lactate dehydrogenase (NADH-LDH) activity in neutrophils of patients with both favorable and unfavorable outcomes exceeded control values in the preoperative period (Figure 2D). After surgery, NADH-LDH activity in patients with a favorable outcome decreased to values consistent with the control range. In contrast, patients with an unfavorable outcome demonstrated persistently elevated NADH-LDH activity at the final stage of observation, with values exceeding both control levels and baseline activity, as well as those observed in patients with a favorable outcome.

It was found that NADH-dependent malate dehydrogenase (NADH-MDH) activity in neutrophils during the preoperative period was elevated relative to the control range only in patients with a favorable outcome of BP (Figure 2E). After surgery, NADH-MDH activity in this group decreased relative to baseline values and returned to the control level. In contrast, in patients with an unfavorable outcome, enzyme activity increased significantly after surgery compared with baseline values, control levels, and activity observed in patients with a favorable outcome.

NADH-dependent glutamate dehydrogenase (NADH-GDH) activity in neutrophils during the preoperative period was elevated compared with control values exclusively in patients with an unfavorable outcome of BP (Figure 2F). Following surgery, enzyme activity in patients with a favorable outcome decreased relative to baseline values, control levels, and activity observed in patients with an unfavorable outcome at the corresponding stage of observation.

The activities of glycerol-3-phosphate dehydrogenase (G3PDH), NADP-dependent malate dehydrogenase (NADP-MDH), NADP-dependent glutamate dehydrogenase (NADP-GDH), and NAD-dependent glutamate dehydrogenase (NAD-GDH) in circulating neutrophils of patients with BP in both outcome groups, before and after surgery, were comparable to control values.

Correlation analysis was performed to examine the relationships between neutrophil phagocytic activity and intracellular dehydrogenase activity. In the control group, the phagocytic number (PN) of the total neutrophil fraction showed positive correlations with the activities of G3PDH (r = 0.38, *p* = 0.038) and G6PDH (r = 0.51, *p* = 0.004). In addition, PN of actively phagocytic neutrophils in this group correlated positively with G6PDH (r = 0.65, *p* = 0.001), NADP-MDH (r = 0.58, *p* = 0.004), and NADH-LDH activity (r = 0.51, *p* = 0.016), whereas PN of weakly phagocytic neutrophils was associated only with NADP-MDH activity (r = 0.46, *p* = 0.033).

In patients with a favorable outcome of BP in the preoperative period, G6PDH activity was correlated with both PI (r = 0.59, *p* = 0.040) and PN (r = 0.65, *p* = 0.029) of the total neutrophil fraction. Moreover, PN of actively phagocytic neutrophils showed positive correlations with G6PDH (r = 0.78, *p* = 0.005) and NADH-LDH activity (r = 0.53, *p* = 0.043), whereas PN of weakly phagocytic neutrophils was positively associated with NAD-dependent malate dehydrogenase (NAD-MDH) activity (r = 0.97, *p* < 0.001) and negatively associated with glutathione reductase activity (r = 0.67, *p* = 0.028). After surgery, phagocytic number values in this group were predominantly correlated with G6PDH activity: PN of the total neutrophil fraction (r = 0.80, *p* = 0.004), PN of actively phagocytic neutrophils (r = 0.78, *p* = 0.005), and PN of weakly phagocytic neutrophils (r = 0.62, *p* = 0.030).

In patients with an unfavorable outcome of DPP in the preoperative period, PN of the total neutrophil fraction showed a positive correlation with NADH-LDH activity (r = 0.51, *p* = 0.004), whereas PN of actively phagocytic neutrophils was negatively correlated with NADH-GDH activity (r = 0.81, *p* = 0.050). In addition, PN of weakly phagocytic neutrophils in this group was negatively associated with NADPH-GDH activity (r = 0.83, *p* = 0.042) and positively associated with NAD-dependent isocitrate dehydrogenase (NAD-ICDH) activity (r = 0.94, *p* = 0.005). No significant correlations between neutrophil phagocytic parameters and intracellular dehydrogenase activity were identified in patients with an unfavorable outcome in the postoperative period.

## 3. Discussion

It has been established that neutrophils are not only active effectors but also highly sensitive indicators of immune-inflammatory activity [25,26]. Under physiological conditions, circulating neutrophils in healthy individuals are considered to remain in a non-activated state until they receive appropriate functional and regulatory signals, such as antigenic stimulation or exposure to cytokines [27,28].

However, in the context of a pronounced systemic inflammatory response, as observed in patients with bacterial peritonitis (BP), neutrophil functional activity undergoes substantial alterations. Moreover, neutrophil reactivity and the intensity of inflammatory processes are capable of mutually reinforcing each other. Thierry and Salmon (2024) demonstrated that inflammation is associated with prolonged and dysregulated neutrophil activation, which in turn sustains and amplifies systemic inflammation [29].

In the present study, we demonstrated that, irrespective of clinical outcome, patients with BP exhibited a marked increase in the proportion of phagocytosing neutrophils compared with healthy individuals, reaching nearly 100%. Phagocytic activity was also significantly enhanced, with a more pronounced increase observed in patients with an unfavorable outcome. On postoperative day 7, the proportion of phagocytosing neutrophils remained high; however, their functional activity diverged markedly depending on disease outcome.

Importantly, increased preoperative neutrophil phagocytic activity in patients with an unfavorable outcome should not be interpreted as evidence of effective immune protection, but rather as a manifestation of excessive and dysregulated inflammatory activation.

Stratification of neutrophils into actively and weakly phagocytic subpopulations revealed substantial differences in their functional activity both before and after surgery. These findings are consistent with current concepts of reverse neutrophil migration from inflammatory foci back into the systemic circulation [30,31], as well as with the role of cytokine-mediated regulation in modulating neutrophil reactivity at the systemic level [32].

According to our data, patients with a favorable outcome of BP are likely to exhibit a higher degree of reverse migration of functionally active neutrophils compared with those with an unfavorable outcome. However, this interpretation remains hypothetical, since reverse neutrophil migration was not directly assessed in the present study [33,34].

In addition, alterations in the functional activity of circulating neutrophils in patients with BP occur in the setting of emergency granulopoiesis, which is accompanied by the release of immature neutrophils with reduced phagocytic capacity into the bloodstream. This phenomenon is particularly pronounced in patients with an unfavorable disease outcome [35].

A growing body of evidence underscores the pivotal role of intracellular metabolism in the execution of neutrophil effector functions [36,37]. It is well established that glycolysis represents the primary energy-generating pathway in activated neutrophils [38,39]. Our findings indicate altered activity of enzymes associated with anaerobic metabolism in the preoperative period, with the most pronounced changes observed in patients with an unfavorable outcome of BP. Nevertheless, these findings should not be interpreted as direct evidence of increased glycolytic flux, since metabolic flux analysis was not performed in this study.

Enhanced glycolytic metabolism in patients with an unfavorable outcome may be attributable, at least in part, to the increased mobilization of immature neutrophils, which are characterized by elevated expression of glycolytic enzymes [35].

Potential interactions between carbohydrate and lipid metabolism in activated neutrophils have been described previously [40,41]. Therefore, we additionally evaluated glycerol-3-phosphate dehydrogenase and NADP-dependent malate dehydrogenase activities as indirect indicators associated with lipid metabolic processes [42,43]. However, these enzymatic measurements do not allow direct conclusions regarding lipid metabolic fluxes.

Completion of phagocytosis largely depends on the generation of reactive oxygen species, which is contingent upon NADPH production via the pentose phosphate pathway [39,44]. The reduced activity of glucose-6-phosphate dehydrogenase observed in patients with BP prior to surgery may therefore contribute to insufficient reactive oxygen species generation and the development of incomplete phagocytosis [17,38,45].

In addition to its role in oxidative burst, NADPH is a critical component of the glutathione-dependent antioxidant system [46]. The postoperative increase in glutathione reductase activity observed in patients with an unfavorable outcome of BP likely reflects activation of antioxidant defense mechanisms in response to intensified lipid peroxidation, which may, in turn, promote neutrophil ferroptosis [47].

Despite the dominant role of glycolysis, mitochondrial metabolism also contributes to neutrophil functional competence [48,49]. A central element of mitochondrial metabolism is the tricarboxylic acid (TCA) cycle, whose intermediates exert important regulatory effects on immune responses [50,51].

The increased activity of several tricarboxylic acid cycle-associated enzymes observed in patients with an unfavorable outcome of BP may reflect persistent alterations in mitochondrial metabolic state. However, direct assessment of mitochondrial respiration and metabolic fluxes was beyond the scope of the present study.

Regulation of substrate redistribution through the TCA cycle is mediated, among other mechanisms, by glutamate dehydrogenase-dependent reactions [52]. The observed changes in glutamate dehydrogenase activity may indicate altered metabolic adaptation of neutrophils in patients with unfavorable outcomes; however, no direct measurements of glutamine synthesis or substrate utilization were performed [53,54].

The level of aerobic energy depends on the state of the hydrogen gradient between the inner and outer mitochondrial membrane, which is maintained by the malate-aspartate shunt with the participation of mitochondrial NADH-MDH [55]. In the present study, increased NADH-MDH activity in patients with unfavorable outcomes may indirectly reflect persistent mitochondrial metabolic activation. These features of changes in NADH-MDH activity once again confirm the maintenance of a high level of aerobic respiration of neutrophils in patients with an unfavorable outcome of BP after surgery.

Thus, the phagocytic activity and metabolic profile of circulating neutrophils are markedly altered in patients with bacterial peritonitis (BP) and display characteristic patterns depending on disease outcome. Both the proportion of phagocytosing neutrophils in the total fraction and their phagocytic activity are substantially elevated during the acute phase of BP. One week after surgery and postoperative therapy, the proportion of phagocytosing neutrophils remains high; however, phagocytic activity diverges according to clinical outcome: it increases further in patients with a favorable outcome, whereas it decreases to control levels in patients with an unfavorable outcome.

The proportions of actively and weakly phagocytic neutrophils in patients with BP are elevated relative to control values and do not differ significantly according to disease outcome. Predictors of an unfavorable outcome include a high phagocytic activity of these neutrophil subpopulations (compared with patients with a favorable outcome) and a postoperative decrease in both cell numbers and phagocytic activity to control levels, accompanied by an increase in the weakly phagocytic fraction. Conversely, in patients with a favorable outcome, the number of phagocytosing cells and the phagocytic activity of actively phagocytic neutrophils increase postoperatively, while the proportion of weakly phagocytic cells declines.

Neutrophil metabolism in BP patients before surgery is characterized by simultaneous activation of anaerobic and aerobic energy pathways, accompanied by reduced glucose-6-phosphate dehydrogenase (G6PDH) activity, which may limit reactive oxygen species production and contribute to incomplete phagocytosis. Postoperative changes in neutrophil metabolism are closely associated with disease outcome. In patients with a favorable outcome, neutrophil energy metabolism (both anaerobic and aerobic) decreases to levels comparable with those of controls. In contrast, patients with an unfavorable outcome exhibit further intensification of anaerobic metabolism while aerobic metabolism remains elevated. In this context, neutrophils from patients with an unfavorable outcome show increased substrate flux towards glutamate and glutamine synthesis, as well as enhanced lipid peroxidation.

These findings indicate that the mechanisms supporting neutrophil phagocytic function differ according to outcome. In patients with an unfavorable course, phagocytosis shifts from a reliance on cytoplasmic NADPH-generating reactions towards mitochondrial metabolic pathways. Based on the observed alterations in neutrophil phagocytic activity and metabolic state one week after surgery and postoperative therapy, it can be inferred that a favorable outcome is associated with effective anti-inflammatory processes within the peritoneal cavity, including the reverse migration of activated neutrophils into the circulation.

## 4. Materials and Methods

### 4.1. Patients and Study Design

The control group consisted of 67 clinically healthy volunteers of a comparable age range. Individuals included in the control group had no clinical signs or history of acute inflammatory, infectious, autoimmune, or chronic decompensated diseases at the time of enrolment. A total of 51 patients with bacterial peritonitis (BP) (23 men and 28 women) aged 30 to 60 years (mean age 49.6 ± 9.4 years) of both community-acquired and nosocomial origin were examined. All patients received treatment in the Department of Purulent Surgery and the Intensive Care Unit of the Krasnoyarsk Interdistrict Clinical Emergency Hospital named after N.S. Karpovich (Krasnoyarsk, Russian Federation).

Baseline severity of the patients’ condition was assessed using the SAPS II score. The initial severity of BP was evaluated using the Mannheim Peritonitis Index and the Abdominal Index. The presence and severity of multiple organ dysfunction were assessed at baseline and dynamically using the SOFA score [21].

The severity of systemic inflammatory response syndrome was evaluated according to the ACCP/SCCM criteria [22].

Depending on the postoperative outcome (8–21 days after surgery), patients were divided into two groups: those with a favorable outcome (*n* = 32) and those with an unfavorable outcome (*n* = 19). Blood samples were collected prior to surgery and on postoperative day 7. The control group consisted of healthy individuals (*n* = 67, mean age 42.6 ± 3.6 years) based on their annual comprehensive medical examination. All individuals in the control group were examined by primary care physicians and underwent standard laboratory tests and chest X-rays.

### 4.2. Assessment of Neutrophil Phagocytic Activity

Neutrophil phagocytic activity was assessed by flow cytometry using FITC-labeled staphylococcal protein A [23]. Conjugation was performed as follows: staphylococcal protein A was dissolved in bicarbonate buffer (pH 9.0), after which FITC (previously dissolved in DMSO to a concentration of 1 µg/mL) was added. The mixture was incubated in the dark for 1 h, washed three times, and adjusted to a final protein concentration of 1 × 10^6^/mL using a turbidity standard.

To 100 µL of heparinized whole blood, 10 µL of FITC-labeled protein A was added, followed by incubation for 30 min at 37 °C. Erythrocyte lysis was performed using a no-wash protocol with VersaLyse reagent (Beckman Coulter, Breya, CA, USA). To quench the fluorescence of FITC-labeled protein A adsorbed on the neutrophil surface, trypan blue solution (0.2 mg/mL) was added to the cell suspension.

Stained cells were analyzed using a Cytomics FC-500 flow cytometer (Beckman Coulter, Breya, CA, USA). At least 50,000 neutrophils were analyzed per sample. The percentage of fluorescent neutrophils (phagocytic index, PI) and the mean fluorescence intensity (phagocytic number, PN, expressed in arbitrary units) were determined. Based on fluorescence intensity, neutrophils were classified as highly or weakly phagocytic.

### 4.3. Neutrophil Isolation and Assessment of Metabolic Activity

Neutrophils were isolated using a standard Ficoll–Verografin density gradient centrifugation method, followed by removal of adherent cells. The purity of the isolated neutrophil fraction was at least 97%, and cell viability ranged from 98% to 100%.

For the assessment of NAD(P)-dependent dehydrogenase activity, 1 × 10^6^ isolated neutrophils were used. Enzyme activity was determined using a bioluminescent method [24]. The activities of glucose-6-phosphate dehydrogenase, glycerol-3-phosphate dehydrogenase, NAD- and NADH-dependent lactate, malate, and glutamate dehydrogenases, NADP- and NADPH-dependent glutamate and malate dehydrogenases, NAD- and NADP-dependent isocitrate dehydrogenase, as well as glutathione reductase, were measured.

Enzyme activity was expressed in enzymatic units per 10^4^ cells (1 U = 1 µmol/min). Measurements were performed using an NAD(P):FMN oxidoreductase–luciferase enzymes preparation derived from *Photobacterium leiognathi* and a biochemiluminescence analyzer BLM-3607 (LLC MedBioTech, Krasnoyarsk, Russia).

### 4.4. Statistical Analysis

Statistical analysis was performed using the Statistica 8.0 software package (StatSoft Inc., Tulsa, OK, USA). Data were described using the median (Me) and interquartile range (IQR, 25th–75th percentiles). Intergroup comparisons were conducted using the Mann–Whitney U test, while within-group dynamics were analyzed using the Wilcoxon signed-rank test. Correlation analysis was performed using Spearman’s rank correlation coefficient. Differences were considered statistically significant at *p* < 0.05.

## 5. Conclusions

Phagocytic activity and intracellular metabolism of neutrophils are markedly altered in patients with bacterial peritonitis (BP) and exhibit outcome-dependent patterns. Preoperatively, the proportion of phagocytic neutrophils of the total fraction and their phagocytic activity are substantially elevated in all patients, whereas neutrophil metabolism demonstrates activation of both anaerobic and aerobic pathways with reduced glucose-6-phosphate dehydrogenase activity, potentially limiting reactive oxygen species production and contributing to incomplete phagocytosis. One week after surgery, the proportion of phagocytosing neutrophils remains high; however, phagocytic activity diverges according to clinical outcome. In patients with a favorable outcome, phagocytic activity of actively phagocytic neutrophils increases further while the proportion of weakly phagocytic cells declines, and energy metabolism normalizes to control levels. In contrast, patients with an unfavorable outcome exhibit a postoperative decrease in neutrophil phagocytic activity to control levels, persistent high aerobic metabolism, enhanced anaerobic activity, increased substrate flux towards glutamate and glutamine synthesis, and intensified lipid peroxidation. These findings indicate that effective postoperative anti-inflammatory processes, including reverse migration of activated neutrophils, are associated with favorable recovery, whereas persistent metabolic activation and oxidative stress in neutrophils correlate with poor prognosis. Functional and metabolic profiling of neutrophils may therefore serve as valuable prognostic biomarkers and provide potential targets for therapeutic modulation of systemic inflammation in BP.

The obtained findings indicate that neutrophil functional activity and enzyme-based metabolic characteristics are associated with postoperative outcomes in BP. However, these results should be interpreted cautiously, since direct metabolic flux analysis and comprehensive immunometabolic profiling were not performed. Further multicenter studies using modern metabolomic and flux-analysis approaches are required to validate the prognostic significance of these parameters.

## 6. Limitations

Several limitations of this study should be acknowledged. First, the study was conducted at a single center with a relatively small sample size, particularly in the subgroup with unfavorable postoperative outcomes, which may limit the generalizability and statistical power of the findings. Second, the observational design does not allow definitive conclusions regarding causal relationships between neutrophil metabolic alterations and clinical outcomes in bacterial peritonitis.

Third, neutrophil functional activity and intracellular metabolism were assessed only at two time points (preoperatively and on postoperative day 7). Therefore, dynamic changes occurring during the early postoperative period or during long-term recovery could not be fully characterized. Serial longitudinal measurements may provide a more comprehensive understanding of neutrophil immunometabolic adaptation in BP.

In addition, the heterogeneity of bacterial peritonitis etiology, severity of systemic inflammatory response, comorbid conditions, antimicrobial regimens, and surgical interventions may have influenced neutrophil function and metabolic activity. Although patients were stratified according to clinical outcomes, residual confounding factors could not be completely excluded.

Another limitation is that the study focused predominantly on circulating neutrophils and did not evaluate local intraperitoneal immune responses, cytokine profiles, neutrophil extracellular trap formation, or molecular signaling pathways associated with immunometabolic regulation. Inclusion of these parameters could provide a more detailed mechanistic interpretation of the observed metabolic alterations.

Finally, while the identified neutrophil metabolic and functional parameters demonstrated associations with postoperative outcomes, their predictive accuracy and clinical applicability as prognostic biomarkers require validation in larger multicenter prospective studies. Further investigations are also necessary to determine whether targeted modulation of neutrophil metabolism may improve outcomes in patients with bacterial peritonitis.

## Figures and Tables

**Table 1:** Phagocytic index and phagocytic number of the total neutrophil fraction in patients with BP before and after surgery (Me, IQR).

Data	Control Values	Patients with Bacterial Peritonitis	
		Favorable Outcome	Unfavorable Outcome
Before surgery			
Phagocytic index (PI), %	54.6 (21.8–89.6)	98.7 (95.7–99.9) *p*_1_ < 0.001	99.4 (96.5–99.9) *p*_1_ < 0.001
Phagocytic number (PN), abs,	57.5 (23.8–114.0)	151.0 (108.0–177.0) *p*_1_ = 0.006	224.0 (156.0–295.5) *p*_1_ < 0.001
After surgery			
Phagocytic index (PI), %	54.6 (21.9–89.8)	97.6 (91.4–99.4) *p*_1_ < 0.001	97.2 (95.4–98.9) *p*_1_ < 0.001
Phagocytic number (PN), abs,	57.5 (23.8–114.0)	326.0 (268.0–356.0) *p*_1_ < 0.001 *p*_i_ < 0.001	113.0 (99.0–127.0) *p*_2_ < 0.001 *p*_i_ < 0.001

Note: *p*_1_—statistically significant differences compared with control values; *p*_2_—differences compared with patients with a favorable outcome; *p*_i_—differences between preoperative and postoperative values.

**Table 2:** Phagocytic index and phagocytic number of actively phagocytic neutrophils in patients with BP before and after surgery (Me, IQR).

Data	Control Values	Patients with Bacterial Peritonitis	
		Favorable Outcome	Unfavorable Outcome
Before surgery			
Phagocytic index (PI), %	36.8 (9.14–48.8)	55.9 (39.8–71.1) *p*_1_ = 0.041	69.9 (51.9–83.6) *p*_1_ = 0.002
Phagocytic number (PN), abs,	113.0 (52.7–205.5)	227.5 (192.0–257.0) *p*_1_ = 0.032	301.5 (243.0–342.0) *p*_1_ < 0.001 *p*_2_ = 0.028
After surgery			
Phagocytic index (PI), %	36.8 (9.1–48.8)	77.11 (70.79–85.10) *p*_1_ < 0.001 *p*_i_ = 0.040	45.41 (35.56–55.25) *p*_2_ < 0.001 *p*_i_ = 0.043
Phagocytic number (PN), abs,	113.0 (52.7–205.5)	382.0 (345.0–439.0) *p*_1_ < 0.001 *p*_i_ = 0.009	184.0 (148.0–190.0) *p*_2_ < 0.001 *p*_i_ < 0.001

Note: *p*_1_—statistically significant differences compared with control values; *p*_2_—differences compared with patients with a favorable outcome; *p*_i_—differences between preoperative and postoperative values.

**Table 3:** Phagocytic index and phagocytic number of weakly phagocytic neutrophils in patients with BP before and after surgery (Me, IQR).

	Control Values	Patients with Bacterial Peritonitis	
		Favorable Outcome	Unfavorable Outcome
Before surgery			
Phagocytic index (PI), %	63.1 (49.4–75.2)	40.4 (28.8–59.2) *p*_1_ = 0.045	32.9 (29.7–46.4) *p*_1_ = 0.008
Phagocytic number (PN), abs,	2.4 (1.9–5.6)	46.2 (37.6–58.3) *p*_1_ < 0.001	45.3 (38.2–48.8) *p*_1_ < 0.001
After surgery			
Phagocytic index (PI), %	63.1 (49.4–75.2)	18.6 (14.8–24.6) *p*_1_ < 0.001 *p*_i_ = 0.002	52.3 (44.3–60.2) *p*_2_ < 0.001 *p*_i_ = 0.039
Phagocytic number (PN), abs,	2.4 (1.9–5.6)	46.6 (37.4–54.8) *p*_1_ < 0.001	54.1 (45.5–62.6) *p*_1_ < 0.001

Note: *p*_1_—statistically significant differences compared with control values; *p*_2_—differences compared with patients with a favorable outcome; *p*_i_—differences between preoperative and postoperative values.

## Data Availability

The original contributions presented in this study are included in the article. Further inquiries can be directed to the corresponding author.
